# Comparative effectiveness of mepolizumab and omalizumab in patients eligible for both: a retrospective cohort study

**DOI:** 10.1186/s13223-026-01055-9

**Published:** 2026-07-16

**Authors:** Tatsuya Nagai, Naoki Inoshima, Hiroki Matsui, Norihiko Kubota, Yoshihiro Funaki, Ayumu Otsuki, Hiroyuki Ito, Kei Nakashima

**Affiliations:** 1 Department of Pulmonology, Kameda Medical Center, 929 Higashi-cho, Kamogawa, Chiba 296-8602 Japan; 2 Department of Clinical Epidemiology and Health Economics, School of Public Health, the University of Tokyo, Tokyo, Japan; 3 Clinical Research Support Office, Kameda Medical Center, Kamogawa, Chiba Japan

**Keywords:** Asthma, Disease exacerbation, Mepolizumab, Omalizumab, Retrospective studies

## Abstract

**Background:**

Omalizumab and mepolizumab are effective biologic therapies for severe asthma. However, their comparative effectiveness in patients eligible for both treatments remains unclear in routine care. Therefore, the aim of this study was to compare the real-world effectiveness of mepolizumab and omalizumab in these patients.

**Methods:**

This retrospective cohort study included patients with asthma who were treated with omalizumab or mepolizumab. Eligible patients had a blood eosinophil count ≥ 150 cells/μL (or ≥ 300 cells/μL within the prior year), total Immunoglobulin E (IgE) ≥ 30 IU/mL, and sensitization to a perennial inhalant allergen. The primary endpoint was the adjusted incidence rate ratios (IRRs) for asthma exacerbation during the first year of treatment. Secondary endpoints were IRRs for emergency room (ER) visits and hospitalizations, subgroup IRRs stratified by baseline eosinophil count (< 300 cells/μL vs. ≥ 300 cells/μL), and change in oral corticosteroid (OCS) dose. Poisson regression models were adjusted for BMI, eosinophil count, and prior-year exacerbations; ER and hospitalization models additionally adjusted for prior ER visits and hospitalizations. Follow-up time was accounted for via an offset term.

**Results:**

We included 49 patients (omalizumab, n = 25; mepolizumab, n = 24). Mepolizumab significantly reduced asthma exacerbations compared with omalizumab (IRR, 0.37; 95% confidence interval [CI], 0.19–0.69). In the subgroup analyses, this effect was significant in patients with baseline eosinophil counts < 300 cells/μL (IRR, 0.38; 95% CI 0.14–0.88) but not in those with baseline eosinophil counts ≥ 300 cells/μL (IRR, 0.47; 95% CI 0.15–1.35). There were no significant between-group differences in the number of ER visits or hospitalizations. The OCS dose was reduced in both groups, with no significant between-group difference in the mean dose reduction.

**Conclusions:**

In patients eligible for both therapies, mepolizumab reduced clinically significant exacerbations more than omalizumab in real-world practice. Although ER visits, hospitalizations, and OCS-sparing were comparable, these findings may help inform biologic selection when exacerbation reduction is prioritized but require confirmation in larger multicenter studies.

**Supplementary Information:**

The online version contains supplementary material available at https://doi.org/10.1186/s13223-026-01055-9.

## Background

Asthma remains a major public health issue, affecting about 300 million people worldwide and causing roughly 1000 daily deaths [1]. Notably, approximately 3.6% of patients are classified as having severe asthma, with symptom control often remaining inadequate despite optimal treatment with inhaled therapies and long-term oral corticosteroid (OCS) [2]. Severe asthma is relatively resource-intensive; furthermore, it results in decreased productivity, which leads to significant physical, social, and economic challenges [3].

Approximately 70–80% of patients with asthma have elevated type 2 inflammation, which is commonly characterized by high eosinophil counts and serum total Immunoglobulin E (IgE) levels [4, 5]. Biological therapies that target type 2 inflammation, including omalizumab (an anti-IgE monoclonal antibody) and mepolizumab (a humanized monoclonal antibody against interleukin-5, which is closely associated with eosinophilic inflammation), can reduce asthma exacerbations, improve symptoms and lung function, enhance quality of life, and decrease OCS requirements [6–9].

These biologic agents are selected based on type 2 inflammatory biomarkers including blood eosinophil counts, serum IgE levels, and evidence of perennial inhalant allergen sensitization [10]. However, patients with multiple elevated biomarkers may be eligible for more than one biologic agent [4, 5]. It remains unclear which biological agent offers the optimal therapeutic benefit for these patients.

Switching from omalizumab to mepolizumab has been shown to improve exacerbation rates and lung function [11]. Another study also showed that mepolizumab may reduce the annual asthma exacerbation rate compared with omalizumab [12]. However, this previous study included patients with blood eosinophil counts ≥ 300 cells/μL, which may have biased the population in favor of mepolizumab. Other studies have reported no significant between-group differences in exacerbation reduction [13, 14]; moreover, there have been no head-to-head trials indicating the most appropriate biologic agent for patients [15].

The aim of this study was to compare the real-world effectiveness of mepolizumab and omalizumab in patients who meet the eligibility criteria for both treatments. Additionally, we evaluated the efficacy of each drug in patient subgroups stratified based on baseline blood eosinophil counts.

## Methods

### Study design and study population

This single-center, retrospective, observational cohort study included patients with asthma aged ≥ 12 years who received omalizumab or mepolizumab at Kameda Medical Center between January 1, 2009, and July 31, 2023. The inclusion criteria were as follows: i) an eosinophil count ≥ 150 cells/μL prior to omalizumab/mepolizumab administration or ≥ 300 cells/μL within the prior year (in patients receiving long-term OCS, ≥ 150 cells/μL was considered sufficient) and ii) serum total IgE levels ≥ 30 IU/mL and a positive result for specific IgE or skin prick testing for at least one perennial inhalant allergen (dust mite, Aspergillus, Alternaria, dog dander, cat dander, moth, or cockroach) [1, 16]. A positive result for specific IgE was defined as a level ≥ 0.35 kU/L [17]. The exclusion criteria were as follows: use of a biologic agent for an indication other than asthma (e.g., eosinophilic pneumonia, allergic bronchopulmonary mycosis, or eosinophilic granulomatosis with polyangiitis) and use of another biologic agent within 1 year prior to treatment initiation. Although previous studies have adopted shorter washout periods [18], we adopted a conservative 12-month washout period to minimize the potential carryover effects from previous biological therapies. The diagnosis of asthma was based on the treating pulmonologist’s clinical judgment documented in the medical records, and all included patients were managed as having severe asthma in our department. Bronchodilator reversibility testing was not systematically performed in all patients, and methacholine challenge testing was not routinely performed at our hospital. The choice of biologic agent was made by the treating physician according to the patient’s overall clinical phenotype, including allergen sensitization, blood eosinophil count, exacerbation history, and treatment availability at the time. The study protocol was reviewed and approved on 17 September 2024 by the Research Ethics Committee of Kameda Medical Center (#24-067). The requirement for informed consent was waived given the retrospective study design and anonymity of patient information.

### Outcomes

The primary endpoint was the incidence rate ratio (IRR) of clinically significant asthma exacerbations within the first year of treatment. The secondary endpoints included the IRRs for exacerbations stratified by baseline eosinophil count (< 300 cells/μL vs. ≥ 300 cells/μL), IRRs for emergency room (ER) visits and hospitalizations, and changes in OCS dosage.

### Definition of acute asthma exacerbation

Based on previous reports and established guidelines, clinically significant exacerbations were indicated by use of systemic steroids for ≥ 3 days or a hospitalization or an ER visit requiring systemic corticosteroid administration [19, 20]. Exacerbations that occurred within 14 days of each other were considered single events.

### Data collection

We collected the following data from the medical records: age; sex; body weight; smoking history; comorbidities (allergic rhinitis, chronic sinusitis, atopic dermatitis, and chronic urticaria); type of inhaled medication; number of asthma exacerbations; corticosteroid dosage; number of hospitalizations and ER visits before and after treatment; and laboratory results such as white blood cell count, eosinophil count, and serum total IgE level.

### Other variables

We performed a subgroup analysis based on the baseline peripheral blood eosinophil count using a cutoff value of 300 cells/μL (baseline < 300 cells/μL vs. baseline ≥ 300 cells/μL), with the treatment effectiveness being assessed separately in each group. Because eligibility also allowed inclusion of patients with eosinophil counts ≥ 300 cells/μL within the prior year, the subgroup with baseline eosinophil counts < 300/μL included some patients with baseline counts < 150 cells/μL.

### Statistical analysis

Given the retrospective and observational nature of this study, we did not perform formal sample size calculation. Instead, we analyzed all available cases. Continuous and categorical variables are presented as means ± standard deviation and percentages, respectively. Between-group comparisons of continuous and categorical variables were performed using the t-test and chi-square test, respectively. IRRs were estimated using Poisson regression with a log-link function. All models were adjusted for baseline body mass index (BMI), blood eosinophil count, and the number of exacerbations within the previous year; furthermore, the ER and hospitalization models were additionally adjusted for baseline ER visits and hospitalizations, respectively. Given the limited sample size, we selected covariates for adjustment based on established predictors. Specifically, we selected the number of exacerbations within the prior year as a predictor of future exacerbations [21], blood eosinophil count as a predictor of treatment response to both biologics [1], and BMI as a risk factor for severe asthma and predictor of treatment response [22]. Additionally, baseline ER visits and hospitalizations were adjusted in the respective models based on prior research [11]. The observation period was defined as up to 1 year after treatment initiation. Patients who switched to another biologic agent during the observation period were censored at the time of switching. For patients who discontinued treatment within 1 year, the data collection period was defined as up to 4 weeks after the last dose. To account for variability in the follow-up duration, we included the natural logarithm of the treatment period as an offset term in all Poisson models. Additionally, we conducted subgroup analyses stratified by eosinophil count to assess differential treatment effects. Within-group post-treatment changes in the rates of asthma exacerbations, hospitalizations, ER visits, and OCS doses were assessed using the Wilcoxon signed-rank test. Furthermore, between-group comparisons of OCS dose reduction were performed using the Mann–Whitney U test. All statistical analyses were performed using R software (version 4.2.1), with a two-tailed *p* value < 0.05 indicating statistical significance.

## Results

### Patient selection

**Fig. 1:**
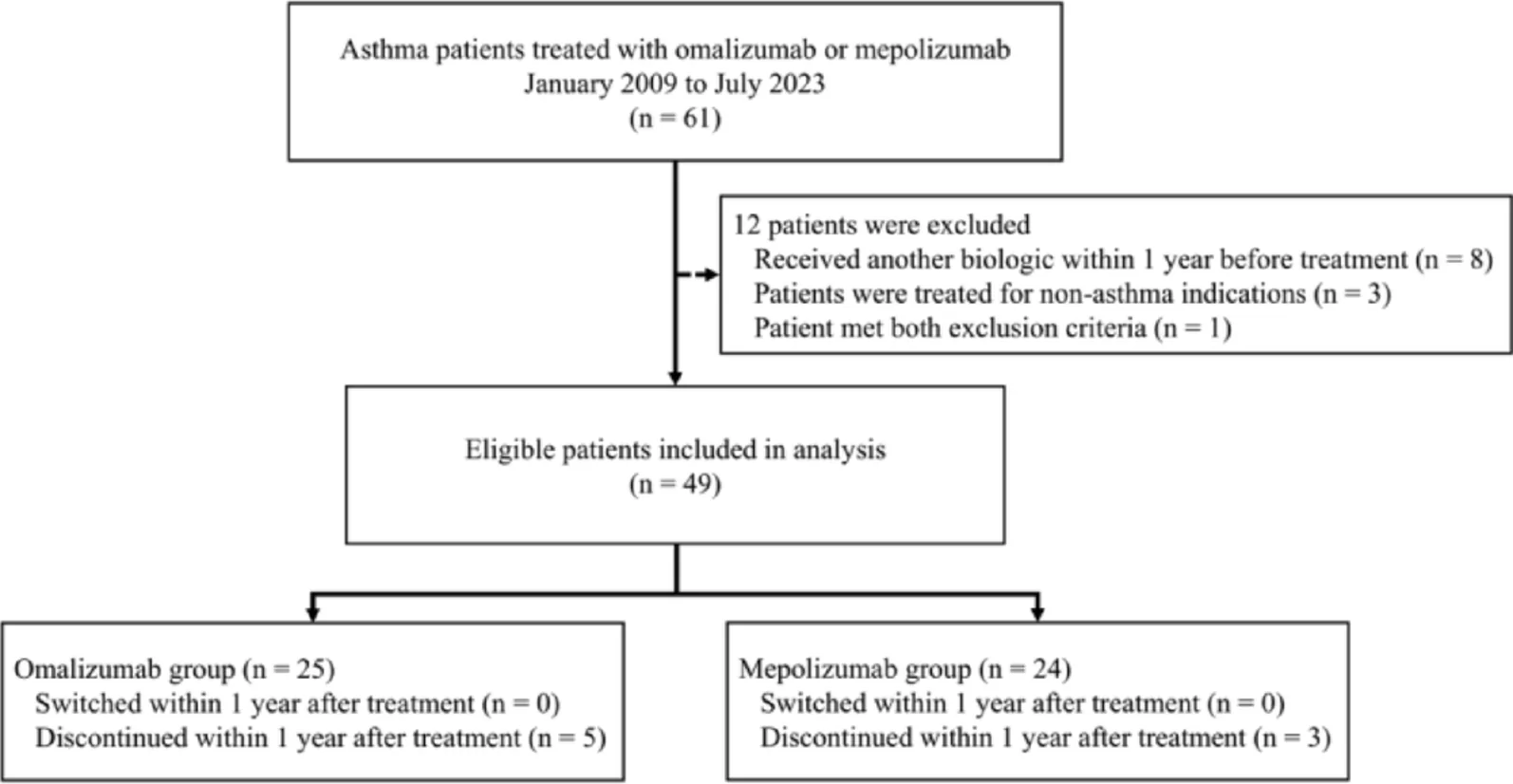
Patient selection flowchart. No patients in either group switched to another biologic within 1 year after treatment initiation. Early discontinuation of the index biologic occurred in five patients in the omalizumab group and in three patients in the mepolizumab group

Figure 1 shows a flowchart of the patient selection process. Among 61 patients who met the initial eligibility criteria, we excluded 8 patients who received other biologic agents within 1 year before treatment initiation, 3 patients who received biologic agents for reasons other than asthma, and 1 patient who met both the exclusion criteria. Finally, 25 and 24 patients were included in the omalizumab and mepolizumab groups, respectively.

### Patient characteristics

Table 1 presents the baseline clinical characteristics of the two groups. There were no significant between-group differences in age, sex, BMI, smoking history, type of inhaled medication, use of OCS, or OCS dosage. However, the omalizumab group had a significantly higher mean number of clinically significant asthma exacerbations and ER visits within the previous year than the mepolizumab group. There were no significant between-group differences in the number of hospitalizations in the previous year. Similarly, there were no significant between-group differences in comorbidities. Although there were no significant between-group differences in the blood eosinophil and maximum eosinophil counts over the past year, serum total IgE levels were significantly higher in the mepolizumab group than in the omalizumab group. Additional diagnostic and follow-up information is summarized in Supplementary Table 1. Documented COPD was present in three patients in each group, and bronchiectasis in one patient in each group. Prior biologic exposure more than 1 year before the index treatment was observed in six patients (25.0%) in the mepolizumab group and in none of the patients in the omalizumab group, whereas no patient in either group switched to another biologic within 1 year following treatment initiation. Eight patients discontinued the index biologic before 1 year (omalizumab, n = 5; mepolizumab, n = 3).

**Table 1:** Baseline clinical characteristics of the study population (before omalizumab or mepolizumab administration)

Variable	Omalizumab (n = 25)	Mepolizumab (n = 24)	*p* value
Age (years)	62.04 (± 14.57)	63.67 (± 19.22)	0.739
Male, n (%)	7 (28.0)	12 (50.0)	0.198
BMI (kg/m^2^)	24.60 (± 5.67)	23.26 (± 2.85)	0.302
Smoking status			0.587
Current, n (%)	1 (4.0)	0 (0.0)	
Former, n (%)	9 (36.0)	10 (41.7)	
Never, n (%)	15 (60.0)	14 (58.3)	
Inhaler			0.153
ICS, n (%)	2 (8.0)	3 (12.5)	
ICS/LABA, n (%)	13 (52.0)	6 (25.0)	
ICS/LABA/LAMA, n (%)	10 (40.0)	15 (62.5)	
Oral glucocorticoid therapy			
Receiving OCS, n (%)	10 (40.0)	12 (50.0)	0.677
Dose in LTOCS users (mg/day, prednisolone equivalent)	10.55 (± 11.07)	6.54 (± 3.45)	0.247
Asthma events in prior year			
Number of exacerbations	4.08 (± 3.58)	1.42 (± 1.72)	0.002
Number of ER visits	1.04 (± 1.43)	0.33 (± 0.56)	0.029
Number of hospitalizations	0.56 (± 0.92)	0.17 (± 0.38)	0.058
Comorbidities			
Allergic rhinitis, n (%)	7 (28.0)	8 (33.3)	0.924
Chronic sinusitis, n (%)	2 (8.0)	4 (16.7)	0.625
Chronic urticaria, n (%)	1 (4.0)	0 (0.0)	1.000
Atopic dermatitis, n (%)	1 (4.0)	1 (4.2)	1.000
Blood test findings			
White blood cell count (cells/μL)	8,792.00 (± 3,448.66)	8,279.17 (± 3308.22)	0.598
Eosinophil count (cells/μL)	428.81 (± 434.14)	606.09 (± 553.88)	0.218
Maximum eosinophil count (cells/μL)^a^	658.17 (± 428.89)	1845.35 (± 3596.11)	0.108
Low eosinophil group (< 300 cells/μL), n (%)	13 (52.0)	10 (41.7)	0.661
Total IgE (IU/mL)	540.59 (± 710.97)	1646.47 (± 2,617.31)	0.047

Continuous variables are expressed as the mean ± standard deviation, and categorical variables are expressed as numbers (%)

BMI: body mass index, ICS: inhaled corticosteroid, LABA: long-acting β-agonists, LAMA: long-acting muscarinic antagonist, LTOCS: long-term oral corticosteroid, ER: emergency room

^a^The maximum eosinophil count was recorded within 1 year before treatment initiation

### Annual mean number of asthma-related events

Table 2 shows the average number of pre- and post-treatment significant asthma exacerbations, ER visits, and hospitalizations. In the omalizumab group, there was a significant decrease in ER visits (p = 0.005) but not exacerbations and hospitalizations. In contrast, the mepolizumab group showed a significant decrease in exacerbations (p = 0.005) but not ER visits and hospitalizations.

**Table 2:** Annual mean number of asthma-related events before and after treatment with omalizumab or mepolizumab

	Omalizumab (n = 25)			Mepolizumab (n = 24)		
	Pre-administration	Post-administration	*p* value	Pre-administration	Post-administration	*p* value
Exacerbations	4.08 (± 3.58)	2.68 (± 3.76)	0.054	1.42 (± 1.72)	0.54 (± 1.02)	0.005
ER visits	1.04 (± 1.43)	0.40 (± 1.08)	0.005	0.33 (± 0.56)	0.12 (± 0.34)	0.109
Hospital admissions	0.56 (± 0.92)	0.24 (± 0.83)	0.098	0.17 (± 0.38)	0.04 (± 0.20)	0.233

*p* values were calculated using the Wilcoxon signed-rank test for within-group comparisons

The median treatment duration was 365 days in both groups

ER: emergency room

### Adjusted IRRs for asthma-related events

**Fig. 2:**
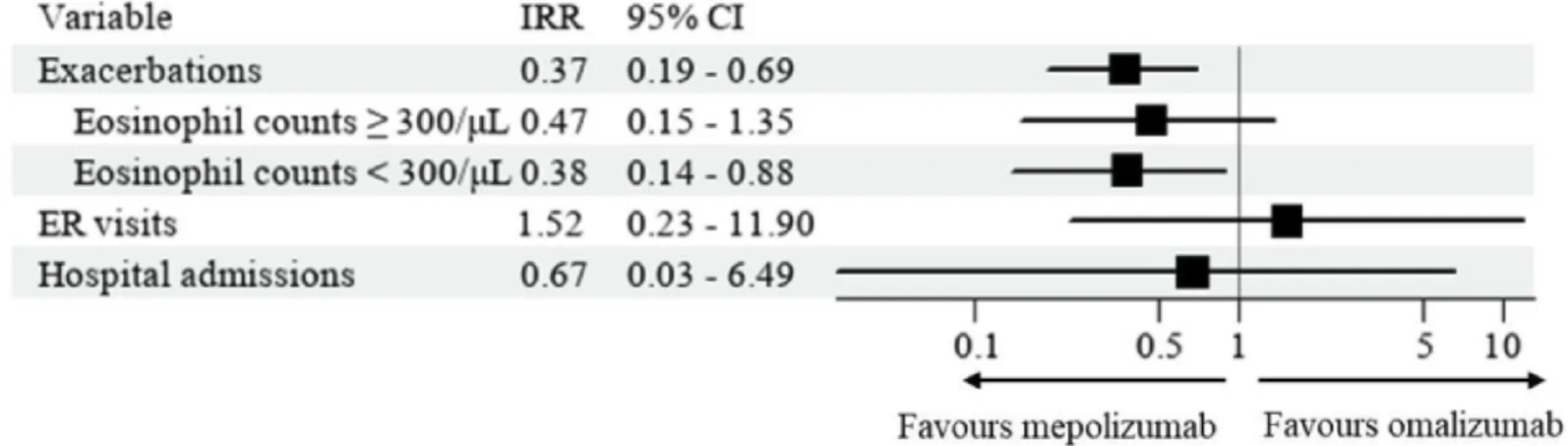
Adjusted IRRs with 95% confidence intervals for asthma-related events, comparing mepolizumab to omalizumab. The incidence rate ratio (IRR) was calculated using a multivariate Poisson regression model. Models were adjusted for baseline BMI, blood eosinophil count, and number of exacerbations within the previous year; additionally, the ER and hospitalization models were further adjusted for baseline ER visits and hospitalizations, respectively

Figure 2 shows the adjusted IRRs from multivariate Poisson regression analysis. The mepolizumab group showed a significant reduction in the annual rate of asthma exacerbations by 63% compared with the omalizumab group (IRR 0.37; 95% confidence interval [CI], 0.19–0.69). In the subgroup analyses stratified by baseline blood eosinophil count, mepolizumab significantly reduced the exacerbation rate in patients with baseline eosinophil counts < 300 cells/μL (IRR 0.38; 95% CI 0.14–0.88) but not in those with baseline eosinophil counts ≥ 300 cells/μL (IRR 0.47; 95% CI 0.15–1.35); however, the point estimate in the baseline eosinophil counts ≥ 300 cells/μL subgroup still favored mepolizumab. There were no significant between-group differences in ER visits (IRR 1.52; 95% CI 0.23–11.90) and hospitalizations (IRR 0.67; 95% CI 0.03–6.49).

### OCS dose reduction

**Fig. 3:**
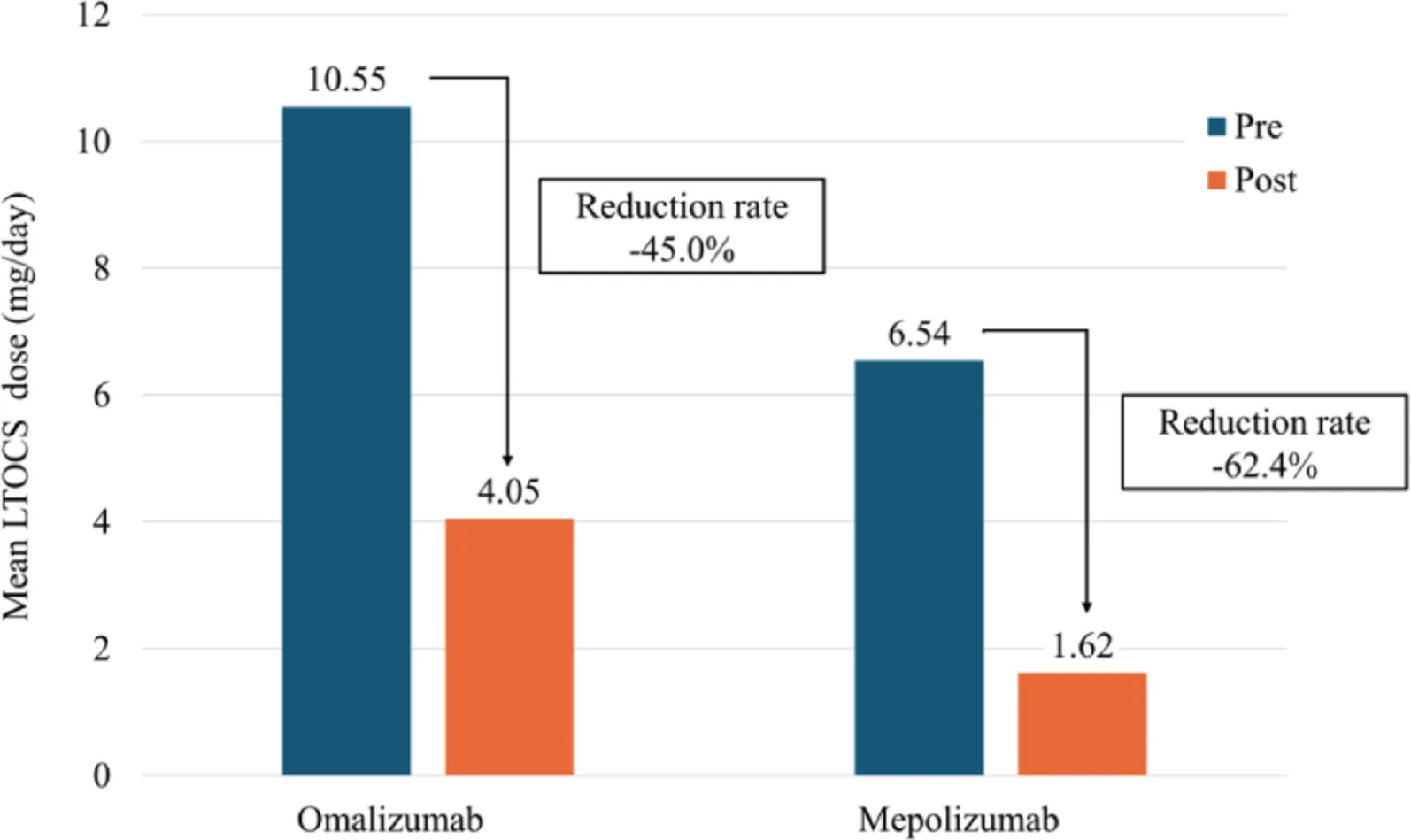
Between-group comparison of the post-treatment reduction in the oral corticosteroid (OCS) dose

Both groups showed a significant reduction in the mean OCS dose (Fig. 3). In the mepolizumab group, the dose decreased from 6.54 (± 3.45) mg/day at baseline to 1.62 (± 1.80) mg/day (p = 0.004). In the omalizumab group, the dose decreased from 10.55 (± 11.07) mg/day to 4.05 (± 5.01) mg/day (p = 0.027). However, there was no significant between-group difference in the magnitude of OCS dose reduction (p = 0.473).

## Discussion

Our findings indicated that mepolizumab significantly lowered the annual exacerbation rate in patients with severe asthma compared with omalizumab. Subgroup analyses revealed that mepolizumab exhibited a significant benefit in patients with baseline eosinophil counts < 300 cells/μL but not in those with counts ≥ 300 cells/μL. However, the point estimate in the ≥ 300 cells/μL subgroup remained below 1.0 (IRR 0.47), suggesting a numerically similar direction of effect despite limited precision. There were no notable between-group differences in terms of the number of hospitalizations or ER visits. Both groups showed significant reductions in the OCS dosage, with no significant between-group difference.

Both omalizumab and mepolizumab are effective biologic therapies for severe asthma associated with type 2 inflammation. Typically, omalizumab is administered to patients with high serum IgE levels and perennial inhalant allergen sensitivity, whereas mepolizumab is recommended for patients with elevated eosinophil counts [1, 23]. However, optimal treatment for patients who qualify for both biologics remains unclear. Previous indirect comparison studies have reported no significant differences in major exacerbations or hospitalizations between these treatments [24]. However, a recent prospective study on patients with eosinophil counts ≥ 300 cells/μL revealed that the annual exacerbation rate was significantly lower with mepolizumab than with omalizumab. Moreover, there were no notable between-treatment differences in the number of ER visits or hospitalizations [12]. Although the population and primary endpoints differed from those of the present study, the EVEREST trial demonstrated that dupilumab was superior to omalizumab in patients with severe chronic rhinosinusitis with nasal polyps and coexisting asthma, supporting the broader concept that non-IgE-targeting biologics may offer greater clinical benefit than omalizumab in selected overlap populations [25]. Our study, which used real-world data from a relatively broader patient population, including those with eosinophil counts < 300 cells/μL, showed that mepolizumab significantly reduced the number of exacerbations.

Our finding that mepolizumab significantly reduced exacerbations—including in patients with eosinophil counts < 300/µL—may be attributed to the variability in peripheral blood eosinophil counts. Approximately 50% of patients with severe asthma show eosinophilic airway inflammation [26]; furthermore, high eosinophil counts have been associated with reduced lung function and frequent exacerbations [27]. Mepolizumab targets IL-5, which is a crucial cytokine that promotes eosinophil growth and differentiation; accordingly, mepolizumab helps lower eosinophil counts, reduce exacerbations, and improve quality of life [9]. However, blood eosinophil levels can be affected by time and environment; moreover, they are not always consistent with tissue eosinophilia [28]. Therefore, mepolizumab may be effective even if a patient's blood eosinophil count temporarily drops below 300 cells/μL. Another possible explanation is that some former smokers in this retrospective cohort may have had coexisting COPD or asthma–COPD overlap that was not fully recognized. Because eosinophilic airway inflammation may represent a treatable trait shared by both asthma and COPD, the favorable response to mepolizumab observed in the subgroup with baseline eosinophil counts < 300 cells/μL may, at least partly, reflect this overlap [29]. This possibility should be taken into account when interpreting the subgroup findings.

Inconsistent with a prospective study [11], we observed a significant benefit of mepolizumab in patients with a blood eosinophil count < 300 cells/μL but not in those with a count ≥ 300 cells/μL. This finding could be attributed to several factors. First, the predictors of response to omalizumab remain somewhat poorly established. In contrast, elevated eosinophil counts (≥ 150 cells/μL) and a history of frequent exacerbations are well-established predictors of response to mepolizumab [30]. Omalizumab targets the Fc portion of IgE and reduces the circulating free IgE levels [10]. However, total serum IgE levels are unreliable indicators of treatment response [31]; furthermore, omalizumab has demonstrated benefits in a wide range of patients, regardless of biomarker profiles [32]. Additionally, post-hoc analyses have suggested that a baseline eosinophil count ≥ 260 cells/μL is a predictor of good response to omalizumab [33, 34]. More broadly, recent biomarker-focused literature has emphasized that even for anti-allergic therapies such as omalizumab, treatment responsiveness may be better predicted by type 2 inflammatory biomarkers, such as blood eosinophils and FeNO, than by total or specific IgE alone [35]. This may have contributed to the relatively greater effect of omalizumab in our high-eosinophil subgroup, and therefore narrowed the efficacy gap with mepolizumab. Second, the small sample size and baseline imbalances between the groups may have contributed to the lack of significant between-group differences. Therefore, the lack of statistical significance in the higher eosinophil subgroup (≥ 300 cells/μL) and the apparent numerical advantage of omalizumab in reducing ER visits should be interpreted cautiously.

Biologic selection in severe asthma is increasingly complex, because multiple agents with different mechanisms of action are now available in clinical practice. Therefore, the present comparison of omalizumab and mepolizumab should not be interpreted as a universal treatment algorithm across all currently available biologics. Rather, the value of this study lies in providing preliminary real-world data in a clinically relevant subgroup of patients who were eligible for both agents. In routine practice, biologic selection was likely influenced not only by patient characteristics, such as allergen sensitization, blood eosinophil count, and exacerbation history, but also by treatment availability at the time. Because omalizumab became available earlier than mepolizumab in Japan, differences in the treatment era may also have contributed to treatment selection. Accordingly, our findings should be regarded as hypothesis-generating and interpreted in the context of the small sample size, retrospective design, and baseline imbalances between groups [36, 37]. Furthermore, recent reviews and guidelines suggest that FeNO may complement blood eosinophil counts in informing biologic selection in severe asthma; however, its role should be interpreted in the context of other biomarkers and clinical features [38]. Unfortunately, FeNO was not systematically collected in this cohort and was available in only four patients, precluding its reliable incorporation into the present analysis.

In our study, both groups showed OCS dose reduction, with no significant between-group difference in the dose reduction. The steroid-sparing effect of mepolizumab was demonstrated in the SIRIUS trial, which included patients with severe eosinophilic asthma [39], and in observational studies evaluating outcomes after switching from omalizumab to mepolizumab [40]. However, real-world studies have shown that both omalizumab and mepolizumab can reduce dependence on OCSs [40]. Notably, there remain no reliable predictive biomarkers for this effect on OCS dependence. This may explain the OCS dose reduction in both groups without a significant between-group difference. Furthermore, the relatively small number of OCS users at baseline, as well as the retrospective design in which tapering strategies were left to the physician’s discretion, may have led to an underestimation of the OCS-sparing effect of mepolizumab.

This study has some limitations. First, this was a small-scale, single-center study, which may have limited the statistical power to detect small between-group differences. In addition, because biologic selection in severe asthma is no longer binary, comparison of only two agents does not provide a comprehensive treatment algorithm for current clinical practice. Second, given the retrospective design, the risk of selection bias could not be ruled out. Objective data supporting asthma diagnosis were not systematically available in all patients. Bronchodilator reversibility testing had not been performed uniformly, and methacholine challenge testing was not routinely available. Therefore, coexisting COPD or asthma–COPD overlap could not be completely excluded, particularly in former smokers, and the subgroup findings should be interpreted with caution. In addition, background inhaled therapy was heterogeneous, and the reasons why LAMA was not added before biologic initiation were not consistently documented. Because the study period spanned different treatment eras, some treatment decisions may also have reflected an earlier clinical era in which LAMA add-on therapy for asthma had not yet been widely incorporated into routine practice in Japan. In addition, some patients discontinued the index biologic before 1 year; although differences in follow-up duration were addressed using an offset term and events were captured for up to 4 weeks after the last dose, heterogeneity in treatment persistence may still have influenced outcome assessment. Third, the omalizumab group had a significantly higher baseline frequency of clinically significant exacerbations and ER visits, which suggests worse disease severity at baseline. Fourth, although we adjusted for potential confounders, including eosinophil count, BMI, prior exacerbation, and hospitalization frequency, residual confounding factors may have affected the results. In particular, secondary outcomes such as ER visits and hospitalizations should be interpreted cautiously because the number of events was small and the confidence intervals were wide. Fifth, we defined sensitization to perennial allergens as a specific IgE level ≥ 0.35 kU/L, which may have led to inclusion of patients with clinically minor sensitization and limited comparability with other studies. Sixth, our cohort comprised patients with total IgE levels > 1500 IU/mL despite these levels being outside the typically approved indications for omalizumab. This may limit the generalizability of our results to populations managed with strict adherence to the prescription guidelines. Finally, FeNO was not systematically collected in this cohort and was available in only four patients, which precluded reliable evaluation of its potential role in biomarker-informed biologic selection. Future research should use multicenter, prospective designs—ideally pragmatic, biomarker-stratified, head-to-head comparisons—to validate these findings and refine biologic selection for overlap-eligible patients.

## Conclusions

In patients eligible for both mepolizumab and omalizumab, mepolizumab may be more effective than omalizumab in decreasing clinically significant exacerbations. Our findings provide preliminary real-world comparative data in patients eligible for both treatments. However, given the small sample size, retrospective design, and baseline imbalances, these findings should be regarded as hypothesis-generating. Further prospective studies incorporating broader biomarker assessment are warranted.

## Supplementary Information

Below is the link to the electronic supplementary material.

Supplementary Material 1

## Data Availability

The datasets used and/or analysed during the current study are available from the corresponding author on reasonable request.

## Abbreviations

IgE: Immunoglobulin E
IRR: Incidence rate ratio
ER: Emergency room
OCS: Oral corticosteroid
BMI: Body mass index
CI: Confidence interval
LTOCS: Long-term oral corticosteroid
