# Supplementary material for: Comparative effectiveness of mepolizumab and omalizumab in patients eligible for both: a retrospective cohort study
Source: Allergy Asthma Clin Immunol. 2026 Jul 16;22:56. doi: 10.1186/s13223-026-01055-9 (PMC13660321; doi:10.1186/s13223-026-01055-9)
Supplement: Supplementary file 1 [file 13223_2026_1055_MOESM1_ESM.docx]

**Supplementary table 1.** Additional diagnostic and follow-up characteristics of the study population.

| Variable | Omalizumab (n = 25) | Mepolizumab (n = 24) | p-value |
| --- | --- | --- | --- |
| Chronic respiratory comorbidities | | | |
| COPD, n (%) | 3 (12.0) | 3 (12.5) | 1.000 |
| Bronchiectasis, n (%) | 1 (4.0) | 1 (4.2) | 1.000 |
| Lung cancer, n (%) | 1 (4.0) | 1 (4.2) | 1.000 |
| Other chronic respiratory comorbidity, n (%) | 0 (0.0) | 2 (8.3) | 0.452 |
| Biologic treatment history / follow-up | | | |
| Prior biologic exposure >1 year before index treatment, n (%) | 0 (0.0) | 6 (25.0) | 0.026 |
| Switched to another biologic within 1 year after treatment initiation, n (%) | 0 (0.0) | 0 (0.0) |  |
| Discontinued index biologic before 1 year, n (%) ^a^ | 5 (20.0) | 3 (12.5) | 0.746 |
| Bronchodilator reversibility testing ^b^ | | | |
| Bronchodilator reversibility testing performed, n (%) | 12 (48.0) | 8 (33.3) | 0.451 |
| Positive bronchodilator reversibility testing, n (%) | 1 (4.0) | 0 (0.0) | 1.000 |

Categorical variables are expressed as numbers (%).

^a^ Among the eight patients who discontinued the index biologic before 1 year, the reasons were adverse event (fatigue), n = 1; worsening of comorbid conditions unrelated to asthma, n = 3; insufficient effectiveness, n = 1; and patient preference, n = 3.

^b^ Bronchodilator reversibility testing was not uniformly standardized, as the required withholding period for inhaled medications could not be confirmed in all cases and the test was not always performed immediately before biologic initiation.
